# Control of motor unit firing during step-like increases in voluntary force

**DOI:** 10.3389/fnhum.2014.00721

**Published:** 2014-09-11

**Authors:** Xiaogang Hu, William Z. Rymer, Nina L. Suresh

**Affiliations:** ^1^Single Motor Unit Lab, Sensory Motor Performance Program, Rehabilitation Institute of ChicagoChicago, IL, USA; ^2^Department of Physical Medicine and Rehabilitation, Feinberg School of Medicine, Northwestern UniversityChicago, IL, USA

**Keywords:** motor unit, firing rate, recruitment threshold, onion skin, rate saturation

## Abstract

In most skeletal muscles, force is generated by a combination of motor unit (MU) recruitment and increases in the firing rate of previously active MUs. Two contrasting patterns of firing rate organization have been reported. In the first pattern, the earliest recruited MUs reach the highest firing rates as force is increased, and later recruited MUs fire at lower rates. When firing rate of multiple MUs are superimposed, these rate trajectories form a concentric layered profile termed “onion skin.” In the second pattern, called “reverse onion skin,” later recruited MUs reach higher firing rates, and crossing of firing rate trajectories for recorded MUs is common (although such trajectories are assembled routinely from different trials). Our present study examined the firing rate organization of concurrently active MUs of the first dorsal interosseous muscle during serial, step-like increases in isometric abduction forces. We used a surface sensor array coupled with MU discrimination algorithms to characterize MU firing patterns. Our objective was to determine whether “onion skin” profiles are contingent upon the force trajectory of the motor task, examined here using step-like increases of force output, and also whether they are manifested at different force levels. Our results revealed that the overall “onion skin” firing rate profile was retained as the force level increased with each force step up to 15% MVC. However, the distribution of firing rates across MUs was compressed with increasing force, and overlapping firing rate of units were observed. This rate compression was largely due to rate saturation of the relatively high frequency discharging MUs. Our results reflect flexible firing patterns across MUs at different levels of excitation drive. It is also evident that many units did not follow all the step increases consistently. This failure to track firing rate increases at higher forces could be due to an intrinsically mediated saturation of firing rates for the low threshold MUs, or potentially to some form of inhibitory interactions between active MUs as the level of excitation of the MU pool is progressively increased.

## INTRODUCTION

Changes of voluntary muscle force are realized by adjustments of both motor unit (MU) recruitment and MU firing rates for units belonging to a given muscle. MU recruitment has been shown to be organized in an orderly manner, in that smaller MUs are recruited earlier and larger MUs are recruited later with increasing excitation. This recruitment rank order is widely known as the “size principle” (Henneman, 1957). Although there are also systematic MU firing rate adjustments in relation to the threshold of recruitment, the specific patterns of firing rate change with increasing voluntary command remain controversial, largely because of conflicting experimental observations.

One potential firing pattern is that earlier recruited units tend to fire slowly, while later recruited MUs fire at higher rates. This form of firing rate organization (termed here the “reverse onion skin” property) shows intersecting rate trajectories with increasing force, and has been reported in both cat (Kernell, 1965; Burke, 1968) and human muscles (Gydikov and Kosarov, 1974; Grimby et al., 1979; Moritz et al., 2005; Oya et al., 2009). This organization is intrinsically appealing because of the hypothetical match of MU firing rate profiles with MU twitch properties. Specifically, earlier recruited MUs tend to have smaller-sized but more prolonged twitches (Milner-Brown et al., 1973), meaning that firing rates can be slower while still maintaining partial fusion of MU forces during repetitive activation.

Conversely, the twitch force profile for later recruited and larger MUs tends to have a shorter duration, with a shorter rise time and a faster decay, which would require a higher MU firing rate for effective fusion of force twitches. Thus in this reverse onion skin scheme, the firing rates would (in theory) be well-matched to the contractile properties of the muscle fibers innervated by the motoneuron, and force output would be maximized for a given set of activated MUs. This strategy would also minimize the fluctuations of muscle force especially at high force levels (Hu et al., 2014b).

The assumption here is that the reverse onion skin pattern is a design feature of the pool that maximizes efficiency and force production, based on the assumed recruitment order of MUs. The firing rates of different MUs would then be expected to be a function solely of the absolute recruitment threshold of the MUs, regardless of the form of force trajectory. However, the firing rate data in those studies reporting this reverse onion-skin pattern were obtained using intramuscular recordings, which are highly selective, yielding few MUs in each trial. As a consequence, earlier studies had to pool results from multiple recording sessions collected at different force levels and even from multiple subjects. This is potentially problematic when making inferences about the MU pool properties.

In contrast, the other firing rate pattern that has been reported is that later recruited MUs tend to fire at lower rates than do earlier recruited MUs, generating a layering effect of the firing rate trajectories over time (termed the “onion skin” property). This scheme has also been reported in both cat (Hoffer et al., 1987) and human muscles (Tanji and Kato, 1973; Freund et al., 1975; De Luca et al., 1982; De Luca and Hostage, 2010) during voluntary contractions. The issue regarding this paradigm is that the later recruited units potentially discharge at an unfused frequency, potentially producing force in an inefficient and fluctuating fashion. One functional benefit regarding this firing organization is that later recruited MUs are more fatigable (Burke, 1967); thus a lower firing rate for these larger MUs could limit fatigue and help maintain a sustained muscle contraction, and could also help fine control of muscle force. Additionally, the lower firing rates in later recruited larger MUs could allow for greater force reserves when needed (De Luca and Hostage, 2010; De Luca and Contessa, 2012).

A majority of the studies that have shown the “onion skin” firing pattern used a ramp-hold task, in which voluntary force is increased slowly followed by a steady hold of the force. However, it is possible that the observed lower firing rates of the higher threshold units recruited close to the end of the ramp force were due to a smaller effective excitation drive, since motor commands necessarily should diminish before the required force transition can take place. However, with further increases of excitatory drive, the initially plateaued firing rate of the later recruited MUs might well increase to a higher rate and potentially surpass the firing rates of the earlier recruited units. In this case, the onion skin pattern is potentially a manifestation of the drive to the motoneuron pool during the single ramp-hold task, and not necessarily a predetermined firing paradigm based on the properties of the motoneuron pool, although the size principle would still determine the order and thus the relative drive to each motoneuron in the pool.

To test this hypothesis, we examined the firing rate organization of concurrently active MUs of the first dorsal interosseous (FDI) muscle during serial, step-like increases of isometric forces. With sequential increases of force levels (excitation drive), we were able to follow the firing rate patterns of the same MUs and quantify the consistency of specific firing patterns at different force levels.

To discriminate MUs, we used a surface electromyogram (sEMG) sensor array coupled with a high-yield MU decomposition algorithm to characterize MU firing patterns. We then relate these firing patterns to recruitment threshold for each unit. The accuracy of the decomposition results for this approach has been assessed previously, and is described in more detail in the Materials and Methods section.

The results reveal that the firing rates of earlier recruited MUs indeed increased further with force level or excitatory drive. However, the overall “onion skin” profile was retained as the force level increased in sequential steps. We also found that the layering pattern was less distinct as muscle force increased, due to a saturation of firing rate of the earlier recruited MUs. Our findings indicate that the “onion skin” profile was retained during different types of isometric contractions including incremental step-like force increases addressed in the current study, as well as for trapezoidal force trajectories examined in earlier studies. The different patterns of firing rate modulation across MUs reflect a flexible firing organization with an increase of the excitation drive to the pool.

## MATERIALS AND METHODS

### PARTICIPANTS

Six right-dominant neurologically intact individuals (three male, three female) volunteered to participate in this study. All participants gave informed consent via protocols approved by the Institutional Review Board under the Office for the Protection of Human Subjects at Northwestern University.

### EXPERIMENTAL SETUP

**FIGURE 1:**
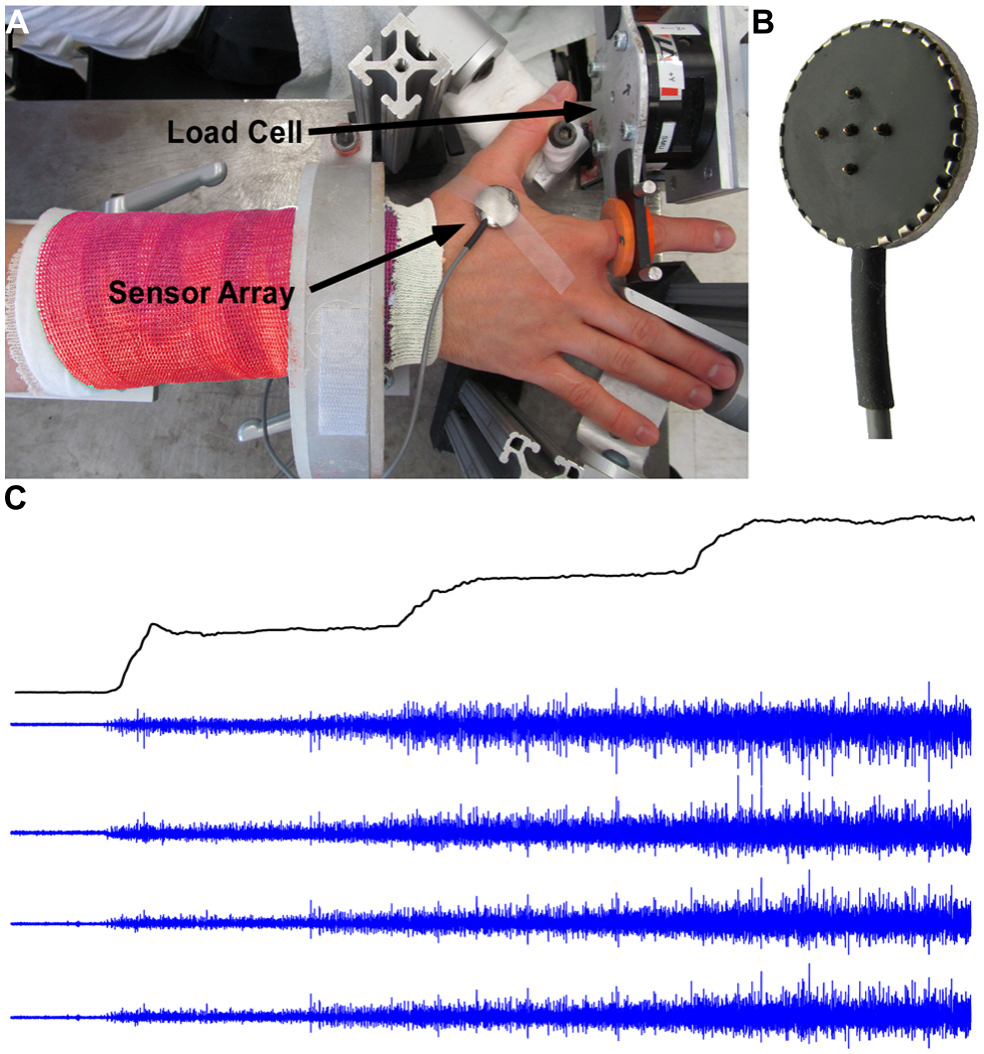
**Experimental setup, force, and EMG signals. (A)** Experimental setup with surface EMG and force signal recordings. **(B)** The five-pin surface EMG sensor array. **(C)** Force trajectory with three force steps, and four channels of surface EMG signals.

Participants were seated upright in a Biodex chair with their upper arm comfortably resting on a support. To standardize hand position and to minimize contributions of unrecorded muscles, the forearm was immobilized with a cast and placed in a ring mount interface attached to a forearm rest. The forearm was placed in full pronation and the wrist was held neutral with respect to flexion/extension. The little, ring, and middle fingers were extended away from the index finger and strapped to the support surface. The thumb was secured at an approximately 60 degree angle to the index finger. The index finger was placed in line with the second metacarpal and the long axis of the forearm creating a 0 degree or neutral metacarpophalangial joint angle (**Figure 1A**). The proximal phalanx of the index finger was fixed to a ring–mount interface attached to a six degrees-of-freedom load cell (ATI, Inc.). The recorded forces from the abduction-adduction direction were low pass filtered (cutoff = 200 Hz) and digitized at a sampling frequency of 2 kHz. The subjects were instructed to produce required abduction forces while minimizing the off-axis forces.

#### EMG recordings

The subject’s skin was sterilized with alcohol pads to ensure proper electric contact and low baseline noise. sEMG was recorded from the FDI using a surface sensor array (Delsys, Inc.) as shown in **Figure 1B** that consists of five cylindrical probes (0.5 mm diameter). The probes are located at the corners and at the center of a 5 × 5 mm square. Pairwise differentiation of the five electrodes yields four channels of sEMG signals (**Figure 1C**). The sEMG sensor and a reference electrode were connected to four channels of a Delsys Bagnoli sEMG system. The signals were sampled at 20 kHz and were amplified and filtered (Butterworth) with a bandwidth of 20 Hz to 2 kHz.

### PROCEDURES

Prior to the main testing session, subjects were asked to perform maximal voluntary contractions (MVCs) for 3 s. This maximum contraction was repeated three times in total, with 60 s rest between trials. The largest value of the three trials was designated as the MVC. The rest of the session consisted of a series of isometric voluntary contractions during which the subject was asked to follow step-like force trajectories displayed on a computer screen. The force output in one exemplar trial is shown in **Figure 1C**. The force trajectory contains four segments: a 3-s quiescent period for baseline noise calculation, a 5% MVC force step (a 0.5-s up-ramp increased at 10% MVC/s, a 9.5-s constant force at 5% MVC), a 10% MVC step (a 0.5-s up-ramp increased at a rate of 10% MVC/s, a 9.5-s constant force at 10% MVC), and a 15% MVC step (a 0.5-s up-ramp increased at 10% MVC/s, a 9.5-s constant force at 15% MVC). Given that the decomposition algorithm is template based and the algorithm works the best in a trapezoid force profile where the action potential template shape is relatively stable. To comply with the algorithm and ensure reliable decomposition results, we limited the force at low levels, such that the force increment at each step is relatively small (i.e., 5% MVC increase per step) and the template shape change is minimal, and meanwhile, the force increment is still large enough to induce measurable changes in firing rate and recruitment of MUs. To ensure that the subjects could follow the force target trajectory closely, they practiced a minimum of five trials of the force steps before the main experiment. For the main part of the experiment, the subjects performed 30 trials with a 60-s rest period between repetitions in order to minimize fatigue.

### DATA ANALYSIS

#### Data processing

The sEMG and force trials were selected for further analysis based on the following criteria:

(a) there was no sudden change (i.e., larger than 20% MVC/s) in the up-ramp force,
(b) the force variability during each steady step was low (within ± 2 standard deviation of background force level), and
(c) the signal to noise ratio >5. The signal to noise ratio was calculated based on the peak–peak amplitude of the baseline noise and peak–peak amplitude of the EMG signal at steady state contractions.

These criteria were based on the suggestions for robust MU discrimination using the dEMG decomposition system (De Luca et al., 2006; Nawab et al., 2010). For each subject, based on the preceding criteria, approximately 10–15 trials were selected for further analysis. The dEMG decomposition algorithm was used to extract single MUs from the EMG data.

For each identified MU, the output from this algorithm consisted of the firing times and four normalized action potential templates from each of the four recorded sEMG channels. Our confidence in this approach is based on prior observations affirming the decomposition accuracy, which has been validated using simulation approaches (Hu et al., 2013a) and a two-source cross-validation method (Hu et al., 2014a). Specifically, in the simulation, we injected random errors to the decomposed spike timing and randomly shuﬄed the decomposed spike trains as well as action potential templates through a surrogate analysis. We found that the perturbed decomposition did not resemble either the action potential templates or the original EMG, suggesting that the original decomposition results were reliable. In the two-source validation, simultaneous intramuscular and surface EMG signals were recorded, and both signals were decomposed independently using separate decomposition algorithms. We found that the decomposition accuracy was 95% on average, based on approximately 120 commonly identified MU pairs from the two types of recordings.

The timing accuracy of the identified MU action potential train was assessed using a spike triggered averaging technique (Hu et al., 2013b), and the validity of the spike triggered averaging has been previously assessed using simulated sEMG signals (Hu et al., 2013c). Specifically, the spike triggered averaging was performed on each of the four channels of the sEMG signals, resulting in four action potential estimates for each MU. The identified firing times for each MU were then used as triggering events for the spike triggered averaging calculation. To ensure reliable estimate of firing rate, we then performed two separate tests to determine which MUs would be retained for further analysis. These tests were designed to assess the stability of the waveform over the trial duration and the degree of match with the decomposition estimated templates.

#### MU recruitment and firing rate estimation

**FIGURE 2:**
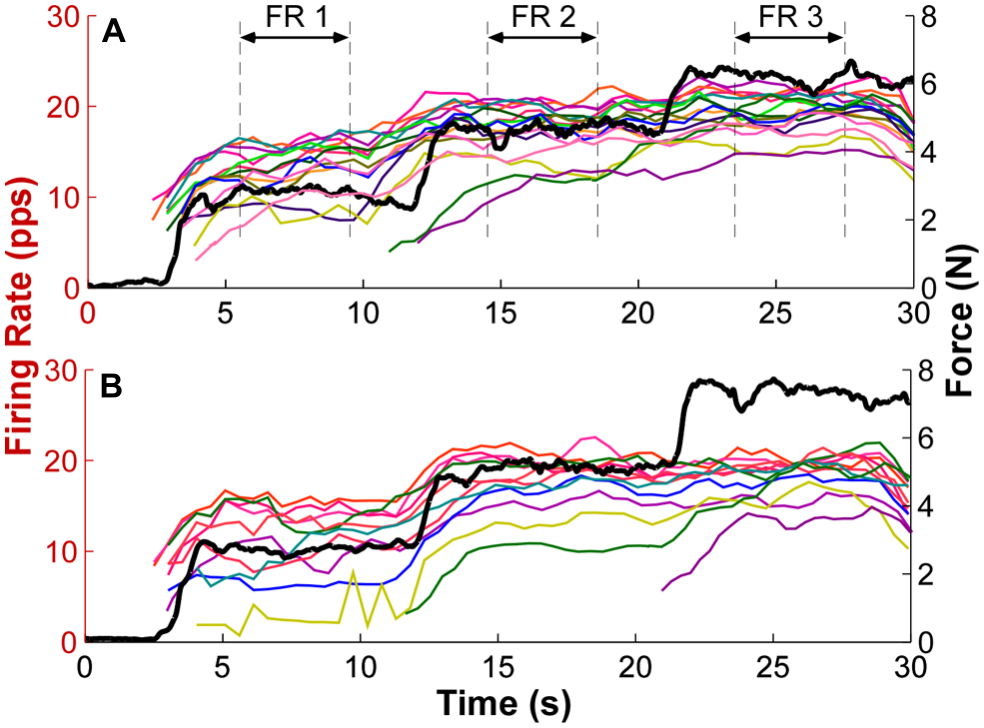
**Exemplar firing rate and force output profiles. (A)** Each thin trace represents the firing rate over time of one exemplar trial. Different colors represent different motor units (MU). The mean firing rate (FR) of a 4-s window as marked by the dashed lines at each force step was calculated as the mean firing rate for each step. The thick black trace represents the force output. PPS represents pulses per second. **(B)** Firing rate and force traces of a second exemplar trial. Additional MUs were also recruited with step increase of force.

To estimate the recruitment threshold, the threshold force of the selected MU was calculated from the averaged isometric force data over an interval (-50 to 150 ms relative to the onset of the first firing event with an inter-spike interval smaller than 300 ms, which was the minimal discharge rate at recruitment). An averaged force was calculated to reduce the influence of force fluctuations registered at the load cell. The window was asymmetric relative to the firing time because of the electromechanical delay (ranging from 30 to 100 ms) between the occurrence of an action potential and a registered force increment (Cavanagh and Komi, 1979; Ce et al., 2013a,b). The mean firing rate was calculated using a 2 s moving window with a step of 0.5 s. The firing rate profiles of individual MUs with overlaid force output from two exemplar trials are shown in **Figure 2**. The mean firing rate (FR1, FR2, and FR3) for each force step was then calculated from a 4-s window as marked by the dashed lines. The middle 4-s window at each step was used because the force was relatively constant at the steady state muscle contraction and the firing rate was relatively stable. In order to track the change of firing rate organization, only the MUs recruited during the first step was used for analysis.

### STATISTICAL ANALYSIS

The organization of MU firing properties as a function of the recruitment threshold was examined at each step increase of force output. A least-squared linear regression between the mean firing rate and the threshold force was performed on the concurrently active MUs at each step. The goodness of fit and the regression slope were compared between the three steps. Given that the regression slope varied considerably between subjects due to different MVC values across subjects, the change of regression slope was compared; specifically, the slope at the first step was used as a reference, and the relative difference between the step 1 and step 2 as well as between step 1 and step 3 were calculated:

(1) $$Change\,\;of\,slope\,=\frac{{Slope}_{i}-{Slope}_{1}}{{Slope}_{1}}\times 100\%\;$$

where *Slope_i_* represents the regression slope at step 2 or step 3, and *Slope*_1_ represents the regression slope at step 1. A negative *change of slope* value means that the regression at step 2 or 3 was shallower than the reference step 1, given that *Slope*_1_ was negative as shown in the Results section.

The mean firing rate and the coefficient of variation (CV; standard deviation normalized by the mean) of firing rate across the concurrently active MUs were also compared cross the three force steps. A one-way repeated measures analysis of variance (ANOVA) was used to test whether the goodness of fit, the change of regression slope, the mean firing rate, and the CV of firing rate differs between force steps. When necessary,*post hoc* pairwise multiple comparisons with Bonferroni’s correction method were used. *P* < 0.05 was considered as statistical significance.

## RESULTS

We recorded surface EMG from the FDI using the array sensor in six intact right-handed subjects. Each trial consisted of a series of step-like increases in voluntary isometric abduction force. Each step sequence provided a substantial body of data, generating typically 10–20 MU recordings that were followed successfully over the step sequence. In total we were able to track several hundreds of units over six subjects.

The firing rate profiles of individual MUs from two exemplar trials are shown in **Figure 2**. Different MUs are represented in different colors, and the force trajectory is also plotted in thick lines. At force step 1, the earlier recruited MU discharge faster and the later recruited discharged slower, forming an “onion skin” pattern. As force increased to higher levels, the initially plateau in firing rate was interrupted, and firing rates increased further. Furthermore, this rate increment was more evident in the later recruited units (e.g., the yellow traces in both panels). As a result, the range of firing rate across units was compressed. Meanwhile, additional MUs were also recruited, and the firing rate of these newly recruited MUs also followed the force steps more closely than the earlier recruited units at step 1. Across the three force steps, the overall “onion skin” layering pattern was retained, although occasional firing rate crossovers were observed.

### FIRING RATE IN RELATION TO THRESHOLD FORCE

**FIGURE 3:**
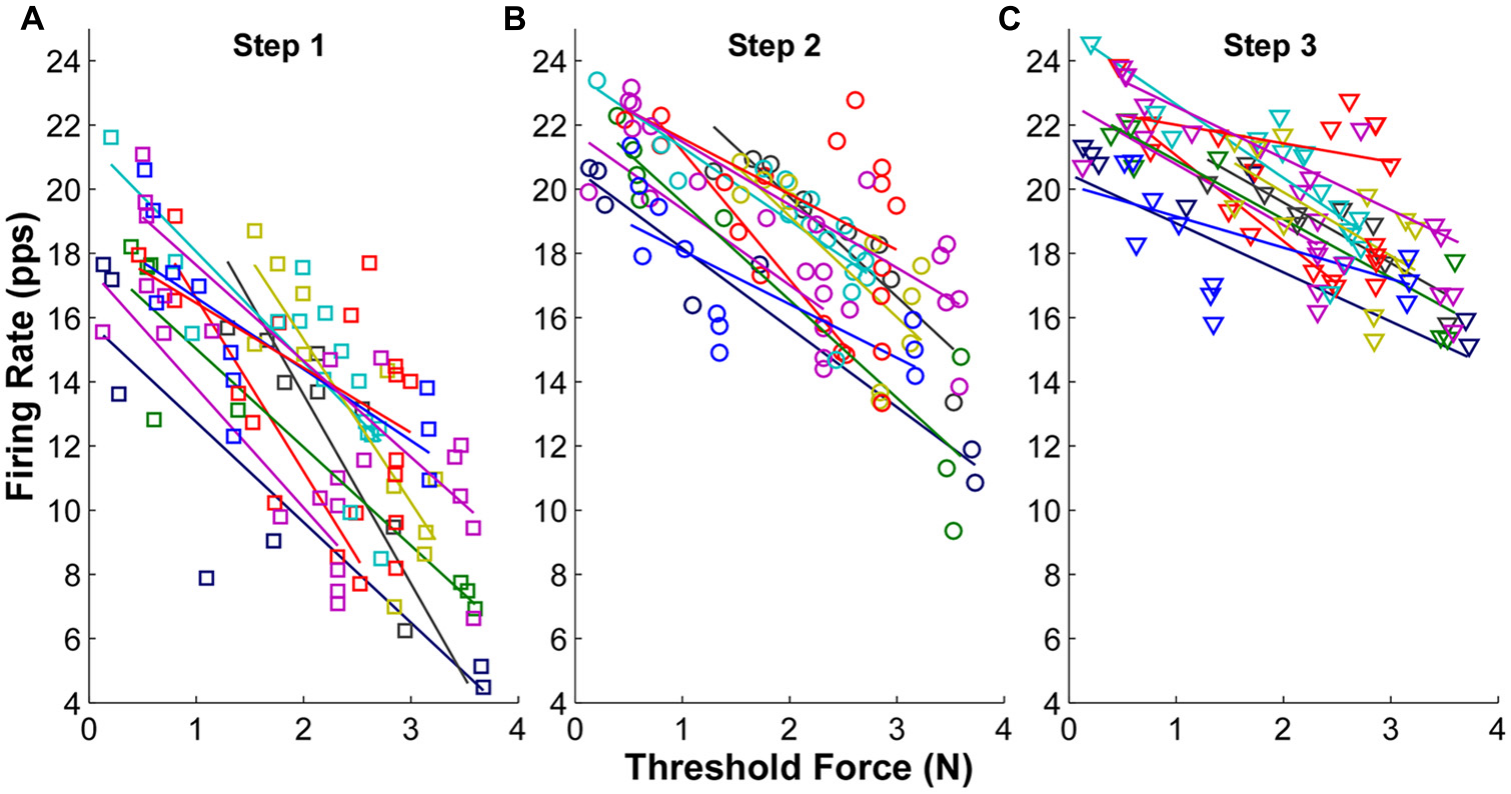
**Mean firing rate as a function of threshold force at each step from one exemplar subject. (A)** Mean firing rate at step 1. Each symbol represents one motor unit, and different colors represent different trials. The linear regression line was also plotted for each trial. **(B)** Mean firing rate at step 2. **(C)** Mean firing rate at step 3.

The firing rate profiles in relation to threshold force at each force step are shown in **Figure 3** for one representative subject. Each symbol represents one MU and the symbols with the same color represent concurrently active MUs from a single trial. When the force was increased voluntarily in sequential steps, the overall firing rate of the recorded MUs increased accordingly. This increment of firing rate was especially evident in later recruited MUs with initially low firing rate. An inverse relation between firing rate and threshold force (i.e., an “onion skin” pattern) was observed consistently across the three force steps. However, as force increased, the rate increments narrowed, and as a result, the regression slope (between firing rate and threshold force) became shallower, largely due to increased firing rates of the later recruited MUs.

**FIGURE 4:**
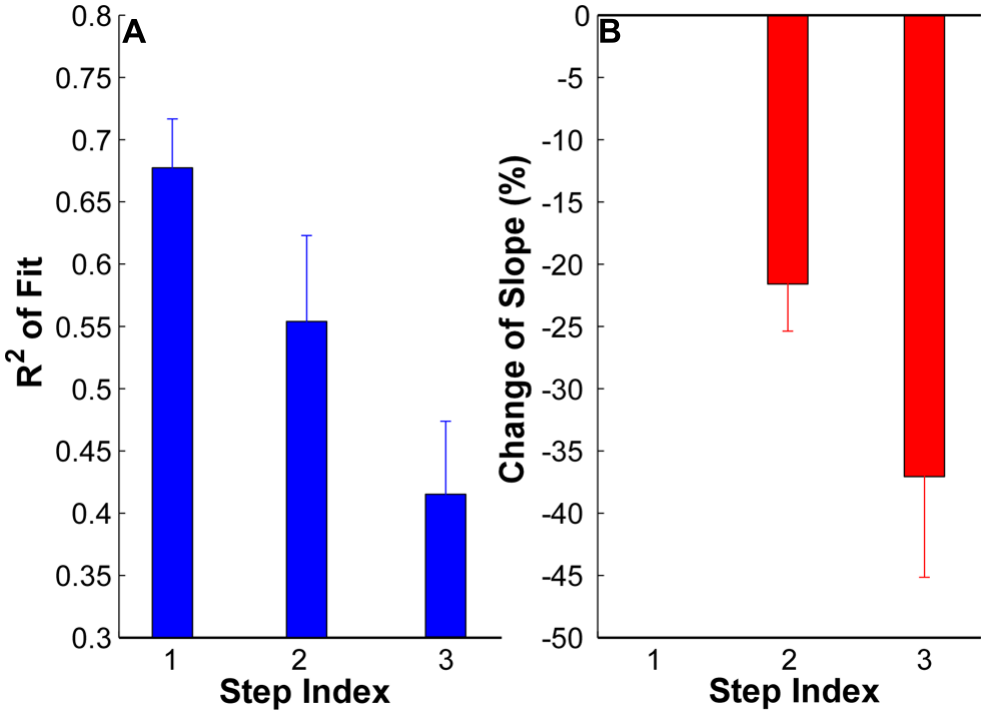
**(A)** Goodness of fit (*R*^2^) at each step of all the subjects. Error bars represent standard errors across subjects. **(B)** Change of regression slope in reference to the first step. The values at step 1 are strictly zero. A negative value here means a shallower slope in step 2 and 3 than in step 1.

The goodness of fit (*R*^2^) plots across the three force steps are summarized in **Figure 4A**. The ANOVA results revealed a significant reduction of *R*^2^ with an increment of force steps (*p* < 0.05) across the whole data set. The *R*^2^ was 0.68 ± 0.03 in step 1, and reduce to 0.55 ± 0.06 in step 2 and 0.41 ± 0.05 in step 3. The reduction of *R*^2^ was significant from step 1 to step 2 and 3 (*p* < 0.05) and was also significant from step 2 to step 3 (*p* < 0.05). Regarding the change of slope (**Figure 4B**), a negative value represents a shallower slope than the reference step 1 [calculated from Equation (1)]. The value at step 1 was strictly zero. The results showed that the regression slope was significantly shallower in step 2 and 3 compared with step 1 (*p* < 0.05). The regression slope in step 3 was also significantly shallower than in step 2 (*p* < 0.05).

### MEAN FIRING RATE AND CV OF FIRING RATE ACROSS MUs

**FIGURE 5:**
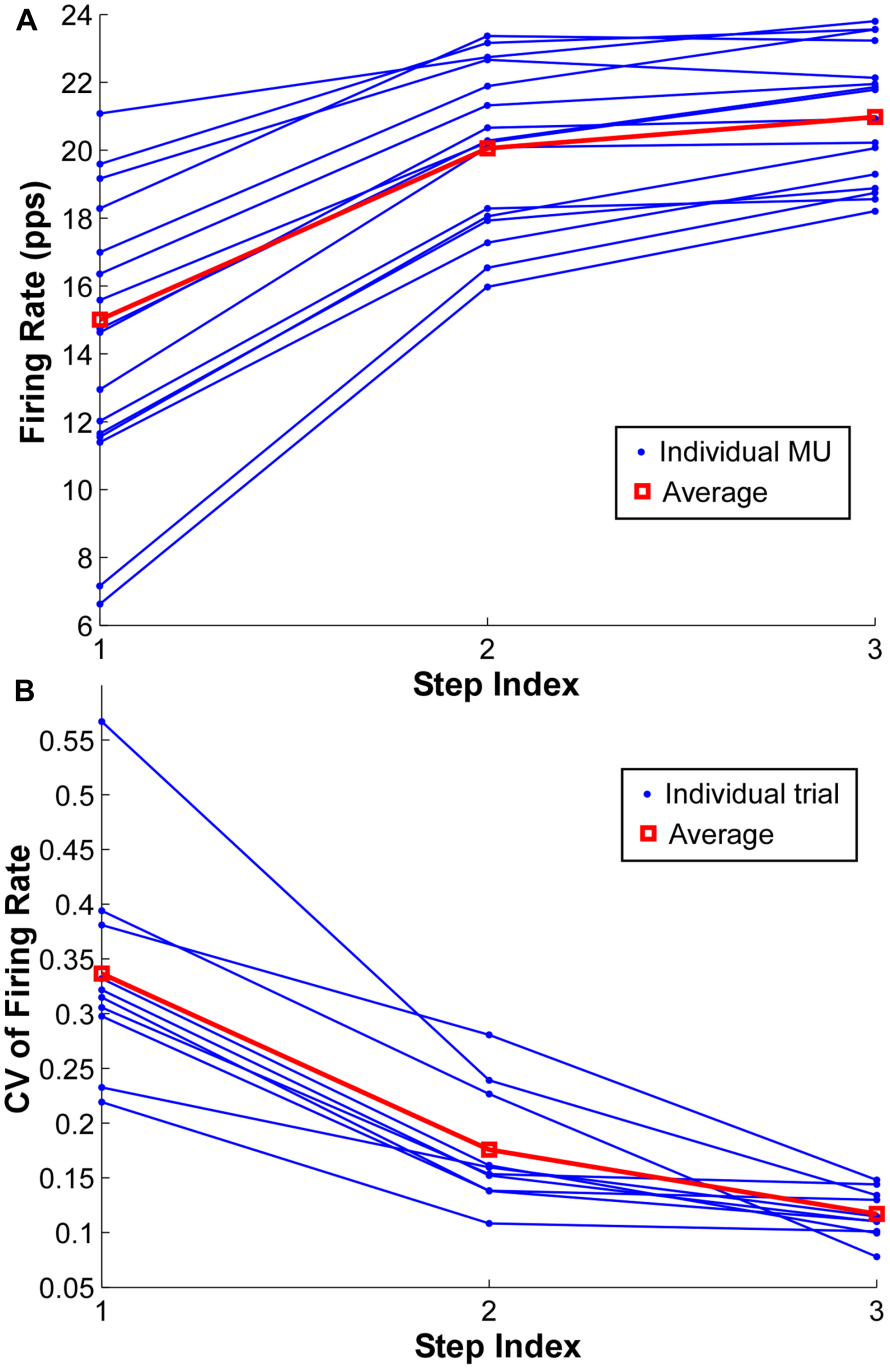
**Firing rate and coefficient of variation (CV) of firing rate at each step. (A)** Mean firing rate at each step of individual motor units in one exemplar trial. The group average is shown as red square. **(B)** CV of firing rate at each step of individual trials from one exemplar subject. One dot represents one trial and the red square represents the group average.

The mean firing rate of the MUs at the three force steps from one exemplar contraction is shown in **Figure 5A**. Each dot represents one MU and the same MU across the three steps is connected by solid lines. The red lines represent the group average from one representative trial. The firing rate increased from step 1 to step 2 consistently across the identified MUs; however, such a rate increment was not evident in most of the MUs with initially high firing rate in the step 1 and 2, and the degree of rate increment in the initially low firing rate MUs was also reduced.

To quantify the compressed range of firing rate with increasing force during a contraction, the CV of mean firing rate across the identified MUs in a single contraction was calculated at each force step (**Figure 5B**). One dot represents the CV from one trial, and the CV from the same trial is connected by solid lines. The red lines represent the group average of one particular subject. As shown in **Figure 5B**, the CV reduced substantially with an increment of force level, especially from step 1 to step 2. However, the CV reduction was not evident from step 2 to step 3 in certain trials.

**FIGURE 6:**
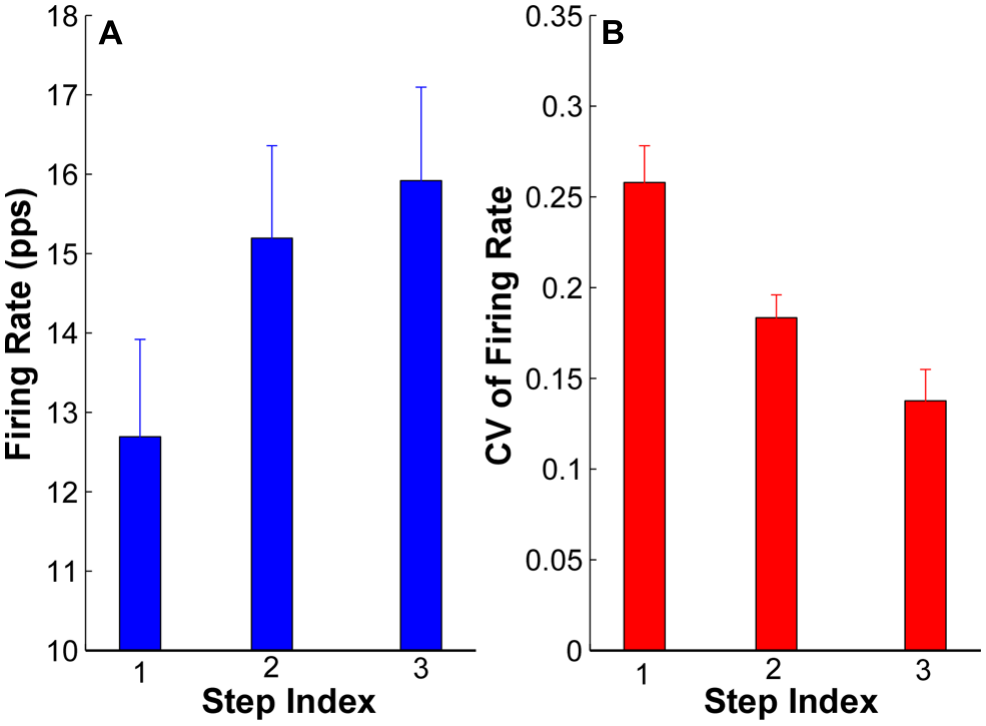
**Mean firing rate and CV of firing rate at each step from all the subjects. (A)** Mean firing rate at each step of all the subjects. Error bars represent standard errors across subjects. **(B)** CV of firing rate of all the subjects.

The averaged firing rate and CV across subjects are summarized in **Figure 6**. The ANOVA results revealed that there was significant increase of firing rate in step 2 (15.19 ± 1.24 pps) and step 3 (16.05 ± 1.23 pps) compared with step 1 (12.69 ± 1.22 pps; *p* < 0.05); and the firing rates in step 2 and 3 were not significantly different (*p* > 0.05). Regarding the CV of firing rate, the CV reduced from 0.26 ± 0.02 in step 1 to 0.18 ± 0.01 in step 2 and 0.14 ± 0.01 in step 3. The reduction of CV was significant from step 1 to step 2 and 3 (*p* < 0.05) as well as from step 2 to step 3 (*p* < 0.05).

## DISCUSSION

This study examined the firing rate organization in relation to recruitment threshold of concurrently active MUs of the FDI muscle during step-like increases of isometric force. We used a sEMG sensor array together with a high-yield MU decomposition algorithm to characterize MU firing patterns in relation to recruitment threshold. The results showed that the “onion skin” firing pattern was retained as the force level was increased in sequential steps and no systematic firing rate crossovers were evident. We did find that the initial plateau in firing rate can be exceeded with these serial steps, and that firing rates of the units can be driven higher with increase of excitatory drive. The further increase of the initial firing rate plateau suggests that the observed onion skin is, at least in part, a consequence of the variation in effective drive to the MUs as a function of motoneuron recruitment threshold. Namely, the earlier recruited MUs received higher effective drive than the later recruited units leading to a higher firing rate observed in the lower threshold units.

We also found the “onion skin” layering pattern was less prominent (i.e., a worse goodness of fit and shallower regression slope) as muscle force increased. The weaker “onion skin” profiles at higher forces was largely due to a compressed range of firing rate in concurrently active MUs (i.e., a reduced CV of firing rate across MUs, due to an increase of firing rate of the later recruited MUs and a minimal increase of firing rate of the earlier recruited MUs). The sequential force steps allowed us to track the firing organization in the same group of active units at different force levels. With an increase of the excitatory drive, the initial plateau in firing rate indeed increased to higher levels. Although the rate increment of the later recruited units was larger, the increment was not large enough to cause systematic crossing of rate trajectories on our rate-time plots.

Given that the force levels tested were up to 15% MVC in the current study (due to the recommended force profiles as described in the Materials and Methods section), more rate crossovers might well be expected at higher force levels, if the excitatory drive is increased to even higher levels. However, we believe this outcome is relatively unlikely, because earlier studies have tested force levels close to maximum effort (De Luca and Hostage, 2010), and a strong “onion skin” firing pattern was still evident.

Two different mechanisms might lead to such a lack of systematic crossovers. First, additional MUs are being recruited during the force ramp, and these newly recruited MUs typically discharge at a low rate, therefore, maintaining the overall layering pattern. Second, as the force ramp is further increased, the later recruited units might eventually plateau at their peak firing rates. In order to confirm the second mechanism, it will be necessary to use a different MU recording technique that is capable of tracking firings from a MU pool over a larger force range than the one currently used in our study.

### “ONION SKIN” vs. “REVERSE ONION SKIN”

Our findings are consistent with earlier reports that have shown concentric “onion skin” firing patterns in either concurrently active MUs (De Luca et al., 1996; McGill et al., 2005; De Luca and Hostage, 2010; De Luca and Contessa, 2012) or in pooled MUs from multiple contractions (Tanji and Kato, 1973). Previous studies have also reported a weaker layering pattern with increasing force. For example, Monster and Chan (1977) showed that there is a consistent concentric layering pattern in steady firing rates from multiple intramuscular recordings, and that the firing rates of later recruited MUs rises at a steeper rate with increasing force, and do eventually catch up and discharge at a rate comparable to the earlier recruited MUs. However, it should be noted that the weaker concentric layering patterns observed in these earlier studies were assessed between different contractions, and thus presumably with different MUs. Our current study was able to extend these findings by tracking the firings of the same active MUs at different force levels during a single contraction. Similarly, with increasing force, a shallower regression slope between firing rate and recruitment threshold has also been reported (De Luca and Hostage, 2010), although again, the degree of change of slope does decline at high force levels (∼50% MVC), which is outside the force range tested in the current study.

Conversely, our current results did not reveal any recordings conforming with the general “reverse onion skin” profile at any force level, although occasional firing rate profile crossings (a key marker of “reverse onion skin” rate profiles) were observed as the force step increased (**Figures 2** and **5**). This “reverse onion skin” firing pattern has been reported in decerebrate animal models, in which motoneurons were activated by tonic muscle stretch, and firing rate profile crossings between MUs were observed (Eccles et al., 1958; Burke, 1968). Similar firing patterns have also been reported during voluntary contractions in different human muscles at different age groups (Moritz et al., 2005; Barry et al., 2007; Oya et al., 2009; Jesunathadas et al., 2012). One common feature of these firing patterns, regardless of the experimental conditions, is that the later recruited MUs tend to show a steeper rise of firing rate as excitation level increases and the rate eventually bypasses the firing rate of the earlier recruited MUs, as the firing rate of the earlier units saturates at rates lower than rates achieved by later units. Whereas in the “onion skin” firing pattern, the firing rate of the later recruited MUs tend to increase in the same or even slower rate compared with the earlier recruited ones.

It is also possible that the two different firing patterns arise from the differences in the MU composition (slow vs. fast) of the muscle; however, both firing patterns have been observed in a large range of muscles with different range of MU types. Specifically, the “onion skin” firing pattern has been observed in the FDI, biceps brachialis, deltoid, tibialis anterior, and vastus lateralis muscles, and the “reverse onion skin” pattern has been observed in FDI, soleus, and tibialis anterior muscles. Therefore, it is unlikely that the range of MU types in a muscle is responsible for to the two different firing patterns.

### MECHANISMS OF LESS DISTINCT “ONION SKIN” LAYERING EFFECT

When the force was increased in sequential steps, the “onion skin” profile became less evident; namely, a poorer goodness of fit and a shallower slope of the linear regression were found at higher forces. The weaker layering effect is largely due to a narrowing of the distribution (i.e., a reduced CV) of the firing rate across MUs. Such a compression of firing rate can arise from a rate saturation of earlier recruited MUs and a relatively large rate increment of later recruited MUs. With increasing force output, presumably an increasing excitatory current input, the later recruited MUs with initially low firing rate have firing profiles followed the force trajectory. This rate increment primarily reduces the range of firing rate across MUs. This large rate increment also leads occasionally to firing rate profile crossovers as shown in **Figures 2** and **5**, which adversely affect the goodness of fit in the linear regression.

Unlike high threshold MUs, the firing rate of many low threshold MUs did not follow all the step increases consistently, especially from step 2 to step 3. This reduced rate modulation could be due to an intrinsically mediated saturation of discharge rates for the low threshold units (e.g., perhaps via persistent inward current (PIC) mechanisms). The PIC is a persistent depolarizing current that amplifies synaptic input. It can trigger an initial steep increase of firing rate, but can also limit the subsequent rate increase due to PIC saturation (Heckman et al., 2005). The reduced rate modulation could also be due to inhibitory interconnections between MUs. Typically, an isolated motoneuron will discharge faster with increasing excitatory current input. However, the recurrent inhibition circuits formed between Renshaw cells, motoneurons, and interneurons generate inhibitory current and can modulate the effectiveness of excitatory current increment (Burke et al., 1971; Hultborn et al., 1979).

In this case, with an increase of excitatory input, the inhibitory input also potentially increases disproportionally, leading to a reduced or unchanged net increase, and therefore rate saturation. Additionally, the high threshold motoneurons are likely to contribute more to the activation of Renshaw cells (Hultborn et al., 1988b) and the amount of inhibitory current received is higher in slower twitch units (Hultborn et al., 1988a). These scaled differences between slow and fast units can contribute to early saturation of firing rate, primarily in the low threshold units.

### ACCURACY OF THE DECOMPOSITION RESULTS

Given that the sEMG decomposition approach is developed recently, it is important to ensure that the decomposed MU results are reliable and that they do reflect physiological properties of the MU pool. Previous studies have evaluated the decomposition accuracy using both simulation approaches (Hu et al., 2013a) and a two-source validation method. Specifically, in the simulation, we introduced random timing noise/errors to the decomposed spike timing and randomly shuﬄed the decomposed spike trains as well as action potential templates through a surrogate analysis. We found that the perturbed decomposition does not resemble the action potential templates or the original EMG signal, when the waveform of action potentials and EMG signals were reconstructed using the perturbed decomposition results, suggesting that the original decomposition results were reliable, at least in the tested force levels up to 50% MVC. We also acknowledge that the simulation approach cannot detest missed firings (false negatives), and the goal of the simulation was to assess the general validity of the dEMG algorithm, rather than assessing the explicit accuracy of particular spike timings. In the two-source validation, concurrent intramuscular and surface EMG signals were recorded, and both signals were decomposed independently using separate decomposition algorithms. We found that the decomposition accuracy was 95% on average in the 119 (10.4%) common MUs out of 1143 identified MUs from the sEMG signals. The two-source method provided critical assessment of the spike timing accuracy detecting both spurious and missed firings; however, the force levels were tested up to 15% MVC. The maximal force level in our current study was also limited at 15% MVC; therefore, the decomposed MU firings are reliable at these force levels.

In addition to the accuracy assessment described above, we performed a spike triggered averaging technique (Hu et al., 2013a) to filter potentially unreliable MU spike trains in the current study. Specifically, we evaluated the stability of the action potential waveform over the trial duration and the degree of match with the decomposition estimated templates, to ensure that decomposed firing train was accurate. However, the dEMG algorithm was developed originally based on a single trapezoid force profile, and a steady state contraction was required to perform the template matching process. It is possible that the series of force steps may induce decomposition errors due to action potential shape changes between force steps, which can provide erroneous firing spike trains. However, this possibility is unlikely because the template tracking algorithm allows a certain degree of progressive change of the action potential shape as in the case of a ramp-up state from 0% up to 90% MVC, and the current study only induced a 5% MVC force change. In fact, our current study shows that the algorithm can detect the increase of MU firing rate with step-increase of force output, indicating that the algorithm does not provide artificially pre-conditioned firing patterns.

## CONCLUSION

In this study, we examined the firing rate patterns of a large number of concurrently active MUs at different force levels during a series of force steps. We found that the initially plateau in firing rate of lower threshold units increased further as excitatory drive is increased; however, the rate increment of discharge in later-recruited units was not strong enough to induce systematic crossings between firing rate profiles. Most importantly, we observed “onion skin” firing profiles across different force levels up to 15% MVC (although the layering pattern was compressed with increasing force). Further study is necessary to examine whether systematic cross-overs between MU firings can occur when higher force levels are tested.

## Conflict of Interest Statement

The authors declare that the research was conducted in the absence of any commercial or financial relationships that could be construed as a potential conflict of interest.
